# CRISPR/Cas9-mediated *GJA8* knockout in rabbits recapitulates human congenital cataracts

**DOI:** 10.1038/srep22024

**Published:** 2016-02-25

**Authors:** Lin Yuan, Tingting Sui, Mao Chen, Jichao Deng, Yongye Huang, Jian Zeng, Qingyan Lv, Yuning Song, Zhanjun Li, Liangxue Lai

**Affiliations:** 1 Jilin Provincial Key Laboratory of Animal Embryo Engineering, College of Animal Sciences, Jilin University, Changchun, 130062, China; 2 College of Life and Health Sciences, Northeastern University, Shen Yang, China; 3 Key Laboratory of Regenerative Biology, South China Institute for Stem Cell Biology and Regenerative Medicine, Guangzhou Institutes of Biomedicine and Health, Chinese Academy of Sciences, Guangzhou, China

## Abstract

Cataracts are the leading cause of vision loss in the world, although surgical treatment can restore vision in cataract patients. Until now, there have been no adequate animal models for *in vivo* studies of artificial lens safety and drug interactions. Genetic studies have demonstrated that *GJA8* is involved in maintaining lens opacity and proper lens development. In this study, a cataract model with *GJA8* gene knockout was developed via co-injection of Cas9/sgRNA mRNA into rabbit zygotes. Our results showed that gene mutation efficiency in the *GJA8* locus reached 98.7% in embryos and 100% in pups, demonstrating that the Cas9/sgRNA system is a highly efficient tool for gene editing in rabbits. In agreement with other studies, our genetic and histology results showed that impaired *GJA8* function caused microphthalmia, small lens size and cataracts. In summary, our novel rabbit model of cataracts will be an important drug-screening tool for cataract prevention and treatment.

Congenital cataracts, characterized by lens opacity, are the leading cause of blindness in childhood^1^. It is estimated that blindness occurs approximately 0.6 to 6 in 10000 infants^2^,^3^. Previous studies revealed that nearly one third of congenital cataracts are caused by genetic mutations^4^, and 18 genes mutations have been confirmed to be related with congenital cataracts^5^. The lens specific gap junction proteins *GJA1*, *GJA3* and *GJA8* play critical roles in transmitting information from highly metabolically active cells to less metabolically active cells. Consequently, these are critical genes in development and function of vertebrate lenses^5^. In fact, GJA8 mutations have frequently been reported to cause congenital cataracts in animals and humans^4^,^6^,^7^,^8^,^9^. *GJA8* encodes connexin50 (Cx50) and has abundant expression in the lens, which is necessary for lens growth and maturation of lens fiber cells^10^,^11^.

In terms of anatomy and physiology, rabbits are more similar to humans than mice or rats, and they have a lower cost maintenance and shorter gestation period than pigs or monkeys^12^. Currently, rabbits have been extensively used as an appropriate animal model in cardiovascular and metabolic disease studies. Additionally, rabbits are also widely used in ophthalmic studies because they have similar eye sizes with humans^13^,^14^.

Recently, the CRISPR/Cas9 system has been extensively used for gene editing in various organisms, such as mice, rabbits, sheep and pigs^15^,^16^,^17^,^18^. The Cas9/sgRNA system uses an RNA-guide Cas9 protein combined with a short RNA (sgRNA), which causes double strand break in the genomic DNA^19^. Additionally, cytoplasmic microinjections of the *in vitro* transcribed mRNA and CRISPR/Cas9 have been successfully used for genome modifications in several mammalian embryos^17^,^20^.

Previous studies have reported that cataracts are commonly caused by dominant gene mutations of *GJA8* in humans^6^,^7^,^9^, *GJA8* knockout mice displayed recessively inherited cataracts^21^, which may be due to poorly understood species differences in *GJA8* inheritance patterns between humans and animals. Because rabbits and humans are similar, the objective of this study was to create a novel animal model that recapitulated human congenital cataracts by using CRISPR/Cas9-mediated *GJA8* gene knockout in rabbits. To evaluate this cataract model, we examined the gene editing efficiency of CRISPR/Cas9, the phenotypes and mutant gene heritability.

## Results

### CRISPR/Cas9-mediated gene targeting of *GJA8* in zygotes

**Figure 1:**
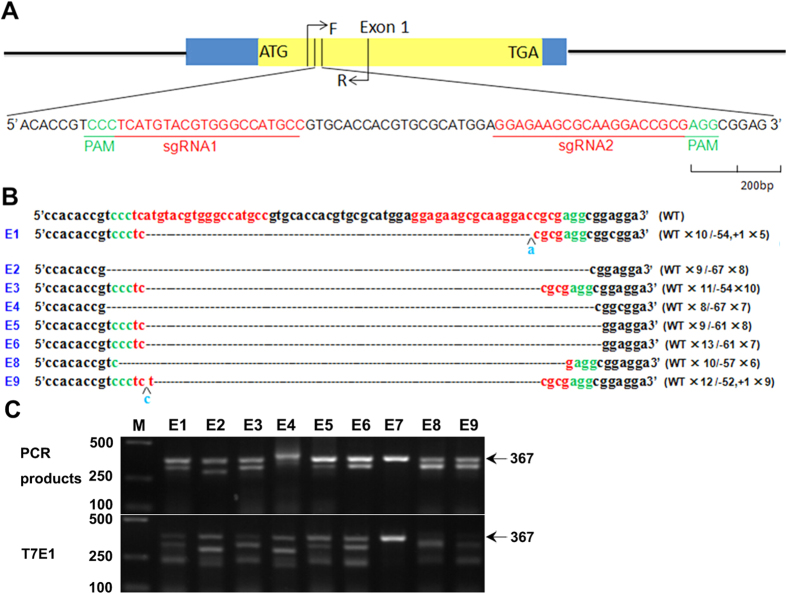
CRISPR/Cas9-mediated gene targeting of *GJA8* in zygotes (**A**) Schematic diagram of sgRNA targeting the *GJA8* gene loci. The yellow rectangle represents the protein coding region of *GJA8*. Two sgRNAs sequences, sgRNA1 and sgRNA2, are highlighted in red. Protospacer adjacent moti (PAM) sequences are presented in green. Primers F and R were used for mutation detection in embryos and pups. (**B**) T-cloning and Sanger sequencing of the modified *GJA8* alleles in blastocysts. Wild type sequence is shown at the top of the targeting sequence. Insertions are highlighted in blue. E: embryos; WT: wild type; deletion “−”; insertion: “+”. (**C**) T7E1 cleavage assay for mutation detection in embryos. Gel images have been cropped. Original images are included in “Authors’ original file for Fig. 1C”. Black arrow indicates the WT allele (367 bp). M, DL2000; E1–E9 represents different blastocysts used in this study.

In order to disrupt *GJA8* in rabbits, two sgRNAs targeting the CDS of *GJA8* were designed (Fig. 1A). To clone the sgRNA sequence into the pUC57-T7-gRNA vector, a *Bbs* I enzyme cut site was added beside the complementary DNA oligonucleotides (Table S1).

To determine the efficiency of the CRISPR/Cas9 system in modifying the *GJA8* gene, *in vitro* transcribed mRNA from Cas9 and sgRNAs was microinjected into the zygote and cultured until blastocyst stage. As shown in Table 1, 85.6% of injected embryos developed to blastocyst stage, indicating that there were no significant differences in cleavage and development rate between non-injected and microinjected embryos. To determine the mutation efficiency, genomic DNA was extracted from a single blastocyst, and the PCR products were sequenced. As shown in Fig. 1B and Table 1, mutation efficiency was as high as 98.7% in the injected blastocyst. This result was confirmed by the T7E1 assay and PCR Sanger sequencing data. Together, this indicated that dural sgRNA directed CRISPR/Cas9 system was efficiently mutated rabbit *GJA8* in our study (Fig. 1C).

### Generation of *GJA8* knockout rabbits via zygote injection

**Figure 2:**
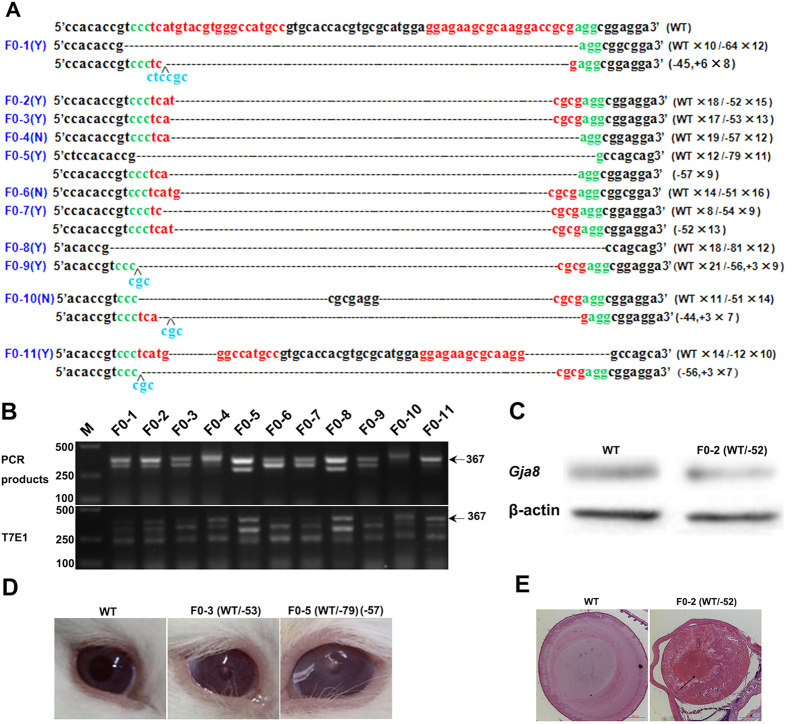
Creation of *GJA8* knockout rabbits via zygote injection. (**A**) T-cloning and Sanger sequencing in 11 pups with modified *GJA8* gene (F0-1-F0-11). Also in “Authors’ original file for Fig. 2A”. sgRNAs sequences are highlighted in red, PAM sequences in green and insertions in blue. WT: wild type; deletion “−”; insertion: “+”. Y: cataract; N: normal. (**B**) T7E1 cleavage assay for mutation detection in F0 generation pups. Black arrow indicated WT allele (367 bp). M: DL2000; F0-1-F0-11 represented the F0 generation pups used in this study. Gel images have been cropped. Original images are included “Authors’ original file for Fig. 2B”. (**C**) Western blots of the *GJA8* gene knockout rabbit lenses. Equal amounts of protein were loaded, and the β-actin was used as an internal control. (**D**) Photographs of mutant founder rabbits. Heterozygous *GJA8* mutant rabbits (F0-3 and F0-5) with cataracts, Other F0 eyes with cataracts were included in Fig. S1B. (**E**) H&E staining of WT and *GJA8* mutant rabbit lenses. The arrows indicate lenses with cataracts. Scale bar, 50 μm.

A total of 52 and 58 injected zygotes (pronuclear stage) were transferred into the oviducts of two surrogate rabbits (Table 2). After 30 days gestation, two recipient mothers gave birth to 11 rabbit pups. Genomic DNA from ears was isolated and used to detect mutations by PCR and sequencing. As expected, all 11 pups had mutated *GJA8*, and the indels in the founders ranged from 12–81 bp deletions (Fig. 2A and Table S2). This result was also confirmed by T7E1 assay, which showed that all F0 rabbits (100%) had the *GJA8* mutation. In addition, genotype data indicated that 45.5% of F0 rabbits had the *GJA8* mosaicism (F0-1, F0-5, F0-7, F0-10 and F0-11), revealing that CRISPR/Cas9 generated mosaicism was commonly detected in F0 animals (Fig. 2B). Since the *GJA8* is mainly expressed in eye lenses, we therefore performed a chimera analysis on lenses from the F0-8 rabbit. As shown in Fig. S1A, the same mutated sequences and cleavage bands were found in between the lenses and ears, suggesting that no chimera mutations were presented in the eyes of *GJA8* mutated rabbit.

We then examined whether the gene mutations reduced of protein levels or changed the cataract phenotype. As shown in Fig. 2C, protein levels were reduced in the lenses of adult *GJA8* (+/−) rabbits when compared to WT counterparts. Photographs showed eight of 11 F0 *GJA8* mutated rabbits appeared to develop cataracts with lens opacities (Fig. 2D, Fig. S1B), when compared to WT littermates. Additionally, the histological H&E staining showed that the lens inner fiber cells of *GJA8* mutants lens were severely distorted (Fig. 2E), compared to the well-aligned inner fiber cells of cataract-free WT rabbits. These observations confirmed that *GJA8* mutations affected lens fiber cells during embryonic lens development and provide a novel animal model that recapitulates human congenital cataracts.

### Off-target analysis in the F0 of *GJA8* gene knockout rabbits

**Figure 3:**
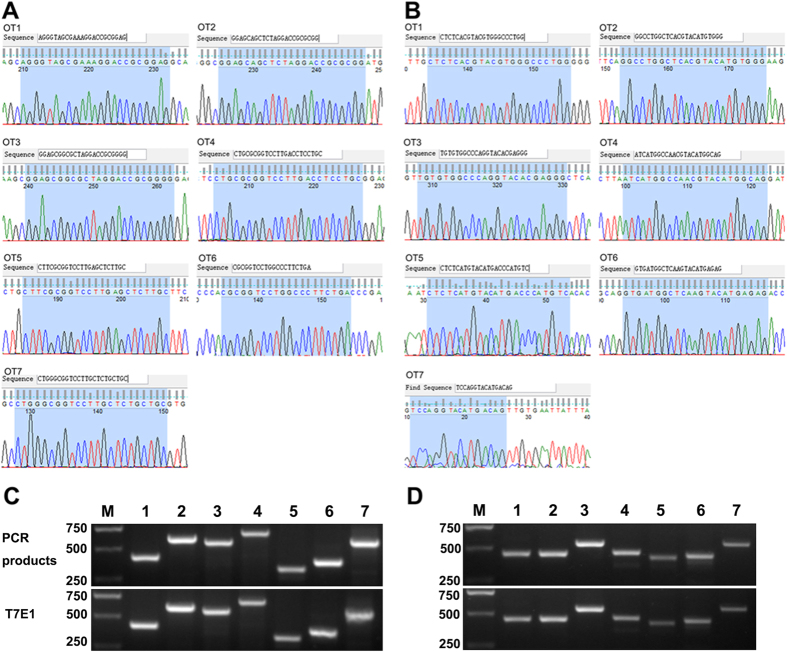
Off target detection in the F0 generation of *GJA8* knockout rabbits (**A**) Chromatogram sequence analysis of seven potential off-target sites (POTS) for sgRNA1 using PCR products in founders. 20 bp of the POTS and the PAM are represented in shadow. (**B**) The chromatogram sequence analysis of seven POTS for sgRNA2 using PCR products in founder rabbits. The 20 bp of the POTS and the PAM are represented in shadow. (**C**) T7E1 cleavage analysis of POTS for sgRNA1. M, DL2000; 1–7 represent the seven POTS. (**D**) T7E1 cleavage analysis of POTS for sgRNA2. M, DL2000; 1–7 represent the seven POTS. Gel images have been cropped. Original images are included in “Authors’ original file for Fig. 3C,D”.

One of the major concerns when using the CRISPR/Cas9 system is off-target mutagenesis, which has been widely reported in human cell lines^22^, mice^20^ and zebrafish^23^. To test whether off-target mutagenesis occurred in the *GJA8* knockout rabbits, we performed Sanger sequencing and the T7E1 cleavage assay on the PCR products from 7 POTS. As shown in Fig. 3A–D, none of the sequencing reads had mutations, suggesting that off-target mutagenesis was eliminated by the co-injection of Cas9 and sgRNAs into rabbit zygotes. The information about POTS was listed in Table S3

### Genotype and phenotype of the F1 *GJA8* knockout rabbits

**Figure 4:**
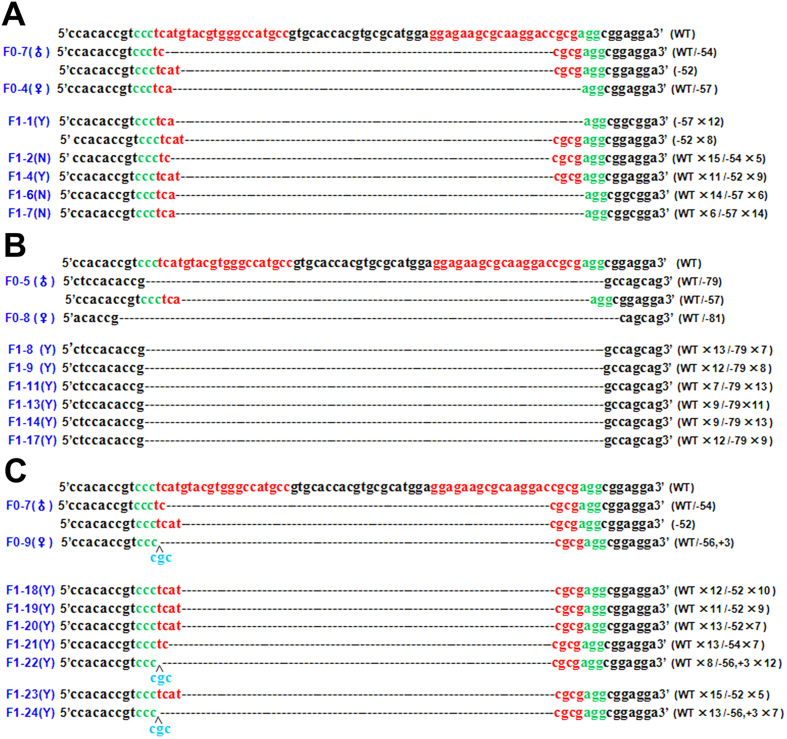
Heritability of the *GJA8* gene knockout rabbits. T-cloning and Sanger sequencing analysis of *GJA8* knockout rabbit pups. (**A**) F0-7 crossed with F0-4. (**B**) F0-5 crossed with F0-8. (**C**) F0-7 crossed with F0-9. sgRNA sequences are highlighted in red, PAM sequences in green and insertions in blue. WT: wild type; deletions “−”, insertion: “+”. Y: cataract; N: normal.

**Figure 5:**
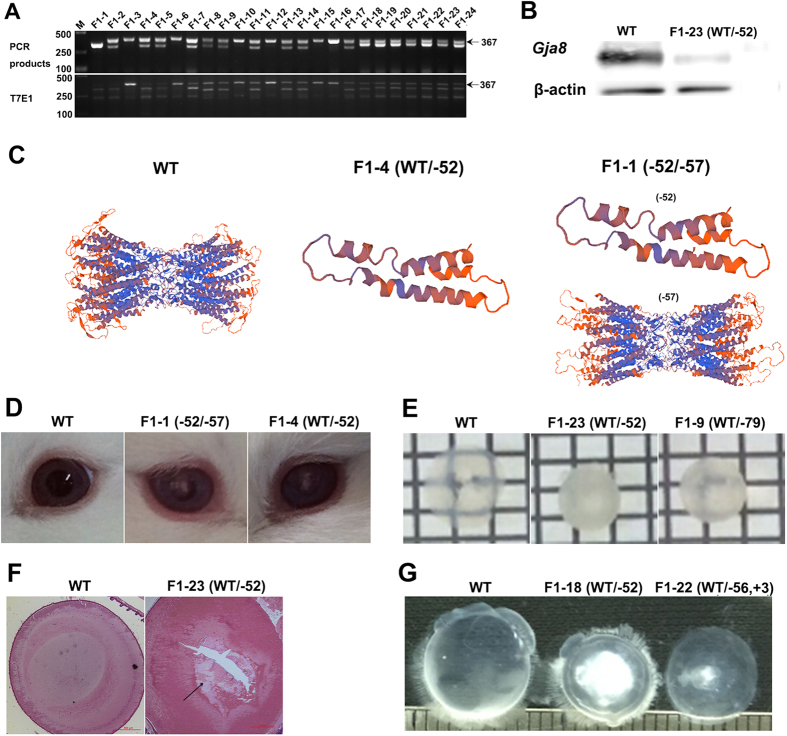
Phenotype identification of the F1 generation *GJA8* knockout rabbits. (**A**) T7E1 cleavage assay for mutation detection in pups. M, DL2000; F1-1-F1-24 represent the offspring used in this study. Gel images have been cropped. Original images have been included in “Authors’ original file for Fig. 5A”. Black arrow indicates the WT allele (367 bp). (**B**) Western blotting from the lens of the *GJA8* gene knockout rabbit. Equal amounts of protein were used and β-actin was the internal control. (**C**) Computer modeling of *GJA8* 3 D structure and impact of the *GJA8* mono-allelic and bi-allelic mutants at the target loci. WT: structure of non-mutant *GJA8* gene; F1-4 (WT/-52): *GJA8* gene with mono-allelic mutation; F1-1 (−52/−57): *GJA8* gene with bi-allelic mutation. (**D**) Phenotypic comparison of eyes and lens between wild type, bi-allelic and mono-allelic mutant (WT, F1-1 and F1-4) F1 generation rabbits at the age of 13 days old. (**E**) Photographs of a *GJA8* mutant rabbit lens with cataract at the age of 13 days old, WT: wild type; F1-23 (WT/−52), F1-9 (WT/−79). (**F**) Histology of the *GJA8* mutant eyes. Histology data from 3 days old wild type and *GJA8* mono-allelic mutant rabbits. (**G**) Photographs of a *GJA8* mutant rabbit lens with cataract at of 3 months old.

To study whether the deletions or indels were heritable, genotypes of the F1 pups (F0-7 × F0-4, F0-5 × F0-8 and F0-7 × F0-9) were determined by PCR and T-cloning Sanger sequencing. As shown in Fig. 4A–C and Table S4, all of the F1 rabbits had the mutation. The F1-1 rabbit was a bi-allelic mutants (−/−) while the others were mono-allelic mutant (+/−). The T7E1 cleavage assay confirmed this result (Fig. 5A). To determine whether the *GJA8* protein had been disrupted, equal amounts of protein from a WT and F1-23 rabbit were used for western blots. As shown in Fig. 5B, *GJA8* protein was detected in the WT but sharply decreased in the F1-23 rabbit. In addition, the predicted 3D models showed *GJA8* protein structure was obviously disrupted in the F1-4 and F1-1 rabbits, compared with WT rabbit (Fig. 5C). Taken together, these data demonstrating the deletions or indels of *GJA8* were inheritable in our cataract rabbit model.

The phenotype of our cataract rabbit model was also examined in the F1 generation. In contrast to WT, *GJA8* (+/−) rabbits showed opacity, smaller lens and obvious microphthalmia (Fig. 5D,E and G and Fig. S1C). Disorganized fiber cell layers were also observed in the founder of our cataract rabbit model (Fig. 5F).

### Disrupted gap junctions in lens fibers of *GJA8* knockout rabbits

**Figure 6:**
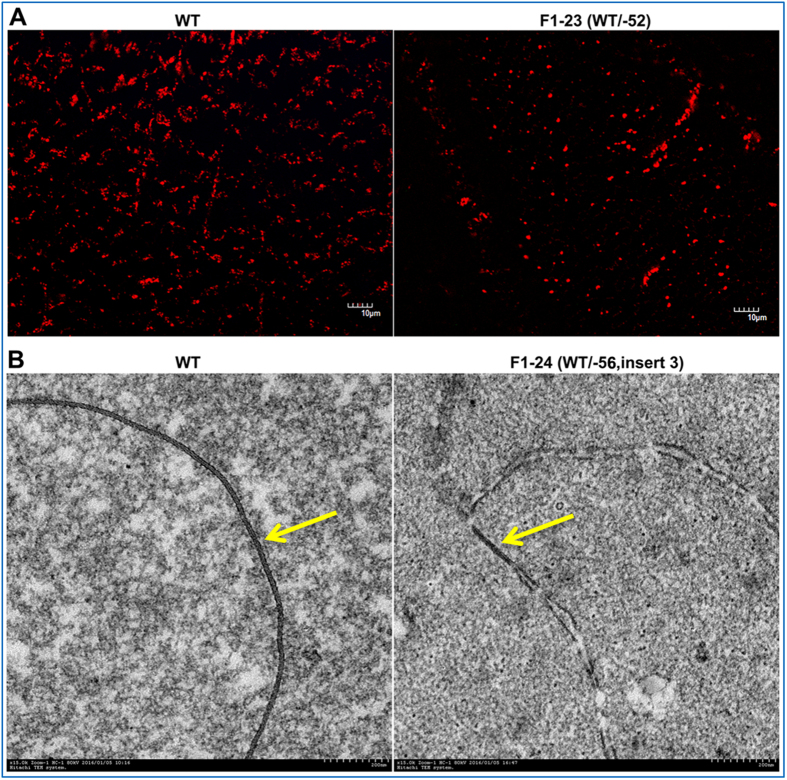
Disrupted gap junction in *GJA8* KO lens fibers. (**A**) Immuolabeling of *GJA8* (red) in lens cortical fibers of paraffin sections from WT and *GJA8* (+/−) rabbits. Scale bar, 10 μm. (**B**) Thin-section of intercellular gap junction in the lens cortical fibers from WT and *GJA8* (+/−) rabbits. Gap junctions are indicated by yellow arrows. Scale bar, 200 nm.

In order to investigate whether gap junctions were affected by *GJA8* mutation, the gap junctions were examined by thin-section immunolabeling and electron microscopy in *GJA8* (+/−) and WT rabbits. According to immunolabeling results, weak fluorescent signals of *GJA8* protein were observed in the outermost fiber cells of F1-23 (WT/-52) rabbit, but stronger punctate signals were seen in WT rabbits (Fig. 6A). Furthermore, the transmission electron microscopy results revealed a much smaller gap junction in the cortical fibers of the *GJA8* mutant rabbit, compared to longer and regular gap junction in the WT rabbit (Fig. 6B).

## Discussion

In this study, we found that the embryo survival and development were not affected by cytoplasmic injections. Also, the efficiency of gene modification was as high as 100% in newborn by co-injection of sgRAN and Cas9 mRNAs into rabbit’s zygotes, which suggesting the cytoplasm injection using CRISPR/Cas9 system is a feasible way to perform gene editing in animals. In addition, Sanger sequencing showed that the indels in the founder rabbits ranged from 12–81 bp. These results suggested that the dual sgRNA directed CRISPR/Cas9 system improved the knockout efficiency, providing a strategy to facilitate gene knockout and large deletions of lncRNA genes^24^. Moreover, CRISPR/Cas9 based cytoplasmic injection has several advantages over traditional methods, like somatic cell nuclear transfers (SCNT), in which abnormal reprogramming in somatic cell clones and low cloning efficiency have been reported in several mammals^25^. Therefore, CRISPR technology shows great promise as a genome editing technique in various vertebrate model systems.

Off-target effects have been described in Cas9-mediated knockout mice^20^ and zebrafish^26^. However, the potential off-target effects were not found in the present study, which could be due to the low concentration (50 ng/μl) of sgRNA and Cas9 mRNAs used. We hypothesized that low concentrations would reduce the off-target effects by causing the sgRNA and Cas9 mRNAs to transiently act on targeting sites and degrade immediately after targeting the gene. In addition, it is particularly important to avoid the mismatches of seed sequences (8–12 bases close to PAM) when design the sgRNA. Furthermore, using cytoplasm microinjection instead of somatic cell nuclear transfer also reduces off-target effects. In fact, to reduce the off-target mutation, the inactivated structural domain of Cas9 (Cas9D10A) or using truncated sgRNAs have been reported to be used in other groups^27^,^28^,^29^,^30^.

Results of the our study revealed that knocking out of the *GJA8* gene in rabbits, which is important for the development and function of vertebrate lens^5^, was sufficient to recapitulate the human cataract phenotype. Previous studies have been reported that the *GJA8* (+/−) mice appeared to have normal eyes and lenses, while only *GJA8* (−/−) mice developed microphthalmia with smaller lenses compared to WT mice^10^. We found, however, that *GJA8* (+/−) rabbits developed cataracts had microphthalmia and smaller lenses. These data were consisted with the clinical data of human *GJA8* cataract phenotypes, due to the dominant gene mutations^5^,^6^,^7^. Our results demonstrated that *GJA8* (+/−) rabbits have dominant cataracts, which was more similar to human cataracts than the mouse in inheritance pattern. Yet, not all these rabbit pups in the F0 or F1 generations exhibited the cataract phenotype. The different lengths of deletion fragments could possibly cause these different lens phenotypes. According to the genotype analysis, cataracts were not observed in rabbits with deletion fragment lengths that were multiples of 3, such as F0-4 (WT/-57bp), F0-6 (WT/-51bp) and F0-10 (WT/-51bp) (−44, +3) rabbits in the F0 generation. These results suggested that *GJA8* transcription was not terminated but produced a mutated protein. The 3D structure indicating that these mutated protein structures of *GJA8* were unstable in the rabbits (Fig. 5C).

In summary, our study showed that knocking out *GJA8* in rabbit via CRISPR/Cas9 system causes human-like cataracts. This novel rabbit cataract model would provide a convenient way to screen new drugs for cataract prevention and treatment.

## Materials and Methods

### Ethical statement

New Zealand rabbits were housed in the Laboratory Animal Center of Jilin University. All animal protocols were approved by the Animal Care Center and Use Committee of Jilin University. All experiments were performed according to the guidelines approved by Jilin University.

### Cas9 mRNA and *GJA8* sgRNA creation

3x FLAG-NLS-SpCas9-NLS was synthesized and cloned into the Pcs2+ vector (Addgene ID 48137). The recombinant Cas9 expression vector was linearized with *NotI*, transcribed *in vitro* by mMessage mMachine SP6 Kit (Ambion) and purified using an RNeasy Mini Kit (Qiagen).

The sgRNAs were designed according to the following website: http://crispr.mit.edu/. Two complementary DNA oligonucleotides were annealed at 95 °C for 5 min to generate double-stranded DNA. Then, DNA was cloned into the *BbsI*-linearized pUC57-T7 vector (Addgene ID 51306). The sgRNAs oligonucleotides sequences targeting *GJA8* were listed in Supplementary Table S1. The recombinant vector (Puc57-T7-sgRNA) was amplified with T7 primers (T7-F: 5′-GAAATTAATACGACTCACTAT A-3′ and T7-R: 5′-AAAAAAAGCACCGACTCGGTGCCA C-3′). The PCR products of gRNA were transcribed using the MAXIscript T7 Kit (Ambion) and purified by with miRNeasy Mini Kit (Qiagen) accroding to the manufacturer’s instructions. Cas9 and gRNA mRNAs quality and concentration were measured by Nandrop 2000 and agarose gel (1.5%) electrophoresis, respectively.

### Microinjection and embryo transfer

The female New Zealand White rabbits (6–8 months old) were superovulated with FSH (60 IU) at intervals of 12 h for 6 times. After the last injection, female rabbits were mated with the male rabbits. Females then received a 100 IU human chorinonic gonadotrophin (HCG) injection. At 18 h post HCG injection, female rabbits were euthanized, and oviducts were flushed with 5 ml DPBS-BSA for zygotes collection. Rabbit embryos at the pronuclear stage (around 18–20 h post-mated) were collected and transferred into oocyte manipulation medium, which contained 9.5 g TCM-199, 0.05 g NaHCO_3_ (Sigma, S4019), 0.750 g Hepes (Sigma, H3784), 0.05 g penicillin, 0.06 g streptomycin, 1.755 g NaCl, 3.0 g BSA, and 1 L Milli Q H2O. A mixture of Cas9 and sgRNA mRNA (200 ng/μl and 50 ng/μl, respectively) was microinjected into the embryo cytoplasm to knock out the *GJA8* gene. The injected embryos were transferred into EBSS medium for short-term culture at 38.5 °C, 5% carbon dioxide and humidity conditions. 30–50 injected embryos were transferred into the oviduct of a recipient rabbit.

### Gene mutation detection in embryos and pups by PCR

The injected embryos were collected at the blastocyst stage. Genomic DNA was extracted with an embryo lysis buffer (1% NP40) at 50 °C for 20 minutes and 90 °C for 5 minutes in a BIO-RAD PCR Amplifier. Genomic DNA from wild type (WT) and *GJA8* knockout (KO) rabbit pups was isolated using the TIANamp Genomic DNA Kit (TIANGEN, Beijing, China). The DNA was amplified with 2×Taq Plus MasterMix (TIANGEN) and PCR primers used to detect mutation were as follows: F- 5′ CGAGAATGTCTGCTACGATGAG- 3′, and R- 5′ CCGGAAACCATACAGGAAGTAG- 3′(Fragment size of *GJA8*-WT allele: 367 bp). The PCR products were purified and cloned into the pGM-T vector (TIANGEN, Beijing, China), and then analyzed by Sanger sequencing. The colonies were picked and sequenced to confirm exact gene mutations.

### Off-target assay

Seven potential off-target sites (POTS) for each sgRNA were predicted to analyze site-specific cleavage by the CRISPR/Cas9 system according to an online design tool (http://crispr.mit.edu/). The PCR products of the POTS were sequenced and confirmed by T7E1 enzyme digestion, as previously described^31^. Primers for POTS determination were listed in Supplementary Tables S2.

### Hematoxylin and eosin (H&E) staining

Eyes tissues from WT and *GJA8* KO rabbits were fixed in 4% paraformaldehyde for 48 h, then embedded in paraffin wax and sectioned for slides. Slides were stained with hematoxylin and eosin (H&E) and viewed under a Nikon ts100 microscope.

### Western blotting

Imediately after rabbits were euthanized, the eyes were ground under liquid nitrogen. The powder of eye tissues was lysed in a protein lysis buffer on ice for 30 min. Protein concentrations were measured using the BCA Protein Assay Kit (Beyotime). Anti-*GJA8* polyclonal antibody (1:2,000; Abcam 199102) was used to measure protein levels. Anti-Beta actin monoclonal antibody (1:2,000; Proteintech 60008-1) was used as an internal control.

3 D structure models of the WT and *GJA8* mutant proteins were built from their amino acid sequences according to the web site: http://swissmodel.expasy.org/ 32.

### Immuno-fluorescence assay

Eye lenses from WT and *GJA8* KO rabbits were fixed in 4% paraformaldehyde for 48 h, embedded in paraffin wax and sectioned for slides. Thin sections were de-waxed with dimethylbenzene and dehydrated using an ethanol gradient (100%, 95% and 80%). The slides were placed in 0.1% Triton solution at 4 °C for 1 h. Slides were blocked with 5% goat serum for half an hour at 37 °C and rinsed with PBS 3 times. Slides were incubated with primary antibody (1:200, Abcam 199102) at 4 °C for overnight. Slides were then incubated with and anti-rabbit secondary antibody (1:500, Invitrogen A-11012) in 5% goat serum for 1 h at room temperature in the dark. Finally, cell nuclei were counter stained with hochest 33342 for 7 min in the dark. Confocal laser microscopy was used to examine the *GJA8* fluorescence.

### Electron microscopy analysis

For morphological analysis by electron microscopy, WT and *GJA8* KO eye lenses were cut into small pieces, and fixed in 0.1 M cacodylate buffer (2% glutaraldehyde, 2.5% formaldehyde, pH 7.2) for 2–4 hours at room temperature. As previous study described^33^, the fixed lenses were rinsed and treated with OsO_4_, tannic acid, and uranyl acetate. Then, they were embedded in Epon. Ultrathin sections were cut with a diamond knife, mounted on copper grids and viewed under an electron microscope (Hitachi H-7650) at 80 Kv.

### Statistical analysis

Percentage for *in vitro* embryo development in the 2-cell, morular and blastocyte groups was compared by chi-square test. A value of *p* < 0.05 was considered statistically significant.

## Additional Information

**How to cite this article**: Yuan, L. *et al.* CRISPR/Cas9-mediated *GJA8* knockout in rabbit recapitulates human congenital cataract. *Sci. Rep.* **6**, 22024; doi: 10.1038/srep22024 (2016).

## Supplementary Material

Supplementary Information

## Figures and Tables

**Table 1:** Cleavage rate of embryos with no-injection (control group) and co-injected with Cas9 and sgRNA mRNAs.

	No. zygotes	2-cell (%)	Morula (%)	Blastocyst (%)	Blastocyst with *GJA8* mutation (%)
Non-injection	30	30(100)	29(96.7)^a^	27(90.0)^a^	0(0)^a^
Injection 1	30	30(100)	27(90.0)^a^	25(83.0)^a^	24(96)^b^
Injection 2	30	30(100)	28(93.3)^a^	26(86.7)^a^	26(100)^b^
Injection 3	30	30(100)	27(90.0)^a^	26(86.7)^a^	26(100)^b^
Total	90	90(100)	82(91.1)	77(85.6)	76(98.7)

^a,b^Values with different superscripts within a column are significantly different.

**Table 2:** Creation of *GJA8*-knockout rabbits via CRISPR/Cas9 system.

Recipients	gRNA/Cas9 mRNA (ng/μl)	Embryos transferred	Pregnancy	Pups obtained (% transferred)	Pups with mutations (%)	Bi- allelic modified (%)	Pups with Cataract (%)
1	40/180	52	YES	5 (9.6%)	5 (100%)	0(0%)	4 (80.0%)
2	40/180	58	YES	6 (10.3%)	6 (100%)	0(0%)	4 (66.7%)
