# Supplementary material for: CRISPR/Cas9-mediated GJA8 knockout in rabbits recapitulates human congenital cataracts
Source: Sci Rep. 2016 Feb 25;6:22024. doi: 10.1038/srep22024 (PMC4766569; doi:10.1038/srep22024)

Supplemental Information

CRISPR/Cas9-mediated *GJA8* knockout in rabbit recapitulates human congenital cataracts

Lin Yuan, Tingting Sui, Mao Chen, Jichao Deng, Yongye Huang, Jian Zeng, Qingyan Lv, Yuning Song, Zhanjun Li, Liangxue Lai

Inventory of Supplemental Information

1. Supplemental Figures

2. Supplemental Tables

3. Original file for Figures

1. Supplemental Figures

Fig S1. (A) Chimeras analysis of the ear and lens from F0-8 rabbit. Black arrow indicates the band of WT allele (367 bp). DL2000, marker. (B) Typical phenotypes of cataracts were determined in founder rabbits of F0-1, F0-3, F0-5, F0-7 and F0-11. (C) Photograph of a cataractous rabbit with *GJA8* mutation at the age of 3 months old.

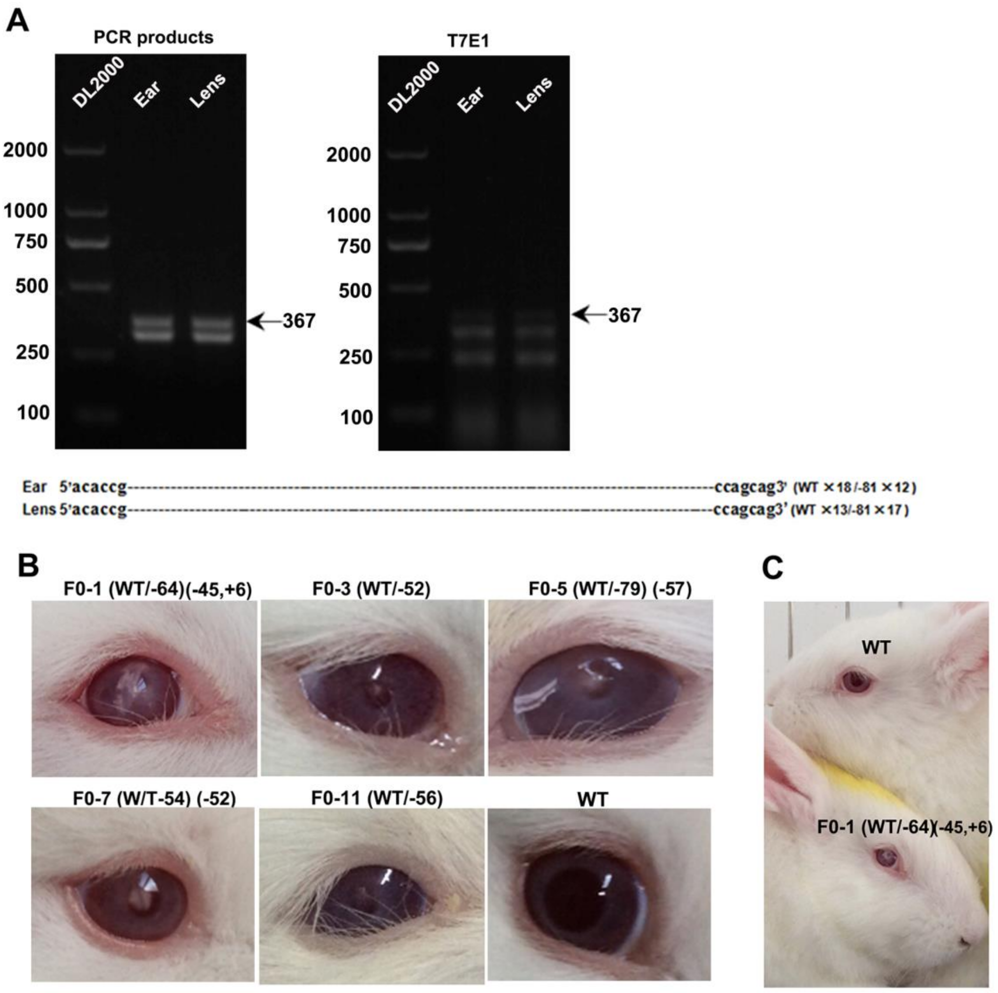

2 . Supplement Tables

Table S1. Two strands of oligonucleotides were used to construct the pUC57-sgRNA vector.

**Target gene**	**Target site**	**PAM**	**Oligonucleotide1**	**Oligonucleotide2**
S1	GGCATGGCCCACGTACATGA	GGG	TAGGCATGGCCCACGTACATGA	AAACTCATGTACGTGGGCCATG
S2	GGAGAAGCGCAAGGACCGCG	AGG	TAGGAGAAGCGCAAGGACCGCG	AAACCGCGGTCCTTGCGCTTCT

Table S2. Alleles from the F0 generation rabbits.

Founder No.	Target site mutations	Sequenced clonies	Phenotype
F0-1	WT	10	+
	Del 64 bp	12	
	Del 45 bp, insert 6	8	
F0-2	WT	18	+
	Del 52 bp	15	
F0-3	WT	17	+
	Del 53 bp	13	
F0-4	WT	19	-
	Del 57 bp	12	
F0-5	WT	12	+
	Del 79 bp	11	
	Del 57 bp	9	
F0-6	WT	14	-
	Del 51 bp	16	
F0-7	WT	8	+
	Del 52 bp	13	
	Del 54 bp	9	
F0-8	WT	18	+
	Del 81 bp	12	
F0-9	WT	21	+
	Del 56 bp; insert 3 bp	9	
F0-10	WT	11	-
	Del 51 bp	14	
	Del 44 bp; insert 3 bp	7	
F0-11	WT	14	+
	Del 12 bp	10	
	Del 56 bp; insert 3 bp	7	

Note: WT: wild type allele; Del: deletion; insert: insertion.

Table S3. Primer sequences of potential off target sites for *GJA8* gene.

	Gene locus	Potential off target sites	Mismatch	Primer sequences (5'-3')
S1	Churn:+250369	AGGGTAGCGAAAGGACCGCGGAG	[1:3:5:10]	F:AGGCACCTCAAGGCATTT<br>R:GTGTATGTCCTTCTCACACCTC
	chr1:+184316759	GGAGCAGCTCTAGGACCGCGCGG	[5:9:11]	F:TAGCGACACAGGTGGGAAA<br>R:GGAGGCTCTACAGGACACTAATC
	chr9:+59430291	GGAGCGGCGCTAGGACCGCGGGG	[5:9:11]	F:GACAATCCAAGTCCACAGAAGT<br>R:AGATGGGAATGGAAAGAAGGC
	chr21-698463	GCAGGAGGTCAAGGACCGCGCAG	[2:5:8:9]	F:CCAAATGCCTGCTTATGTTTCT<br>R:GGGTCCTTATAGGAGGAAGTTAGA
	chr12-142395190	GCAAGAGCTCAAGGACCGCGAAG	[2:4:5:9]	F:CCAAATGCCTGCTTATGTTTCT<br>R:CTTCTGTTTCCTCCCTCTGATG
	chrUN0:-10369	TCAGAAGGGCCAGGACCGCGCGG	[1:2:8:11]	F:GGACTAGGAATAGCTCAACAAGAA<br>R:GGCAGCACAATGACGATTTAC
	chr16-20577150	GCAGCAGAGCAAGGACCGCCCAG	[2:5:8:20]	F:TCGCTCACCTGCCTCGGCGACG<br>R:GTTCTGGAACTAAGAGGCCAC
S2	chrUN0:-304499	CCCAGGGCCCACGTACATGAGAG	[1:2:5]	F:GCTCTGCACAGTGACCTTTA<br>R:CCTTCCCAGGCAAGAGTTATC
	chr1:+90977471	GGCCTGGCTCACGTACATGTGGG	[4:9:20]	F:CAAGAGTCCAACCACGTCTT<br>R:TTCTGGGAAATGATCCGTGATAG
	chrUN0:+885137	TGTGTGGCCCAGGTACATGAGGG	[1:3:4:12]	F:CGAGAGCAGGAAGCTCATAAAG<br>R:CAGACTTGTGCTGTGAGTGTAG
	chr7:+155044047	ATCATGGCCAACGTACATGGCAG	[1:2:10:20]	F:GTAGAAAGCAGTCTGGCCTATG<br>R:GGATGTTGAGGCAGTGAGTATATTA
	chr4:-45197482	GACATGGGTCATGTACATGAGAG	[2:8:9:12]	F:TGCTTCCAGTTATGGGTGTTAT<br>R:TTCACTATCCAATCTGCCTGTC
	chr1:+126065974	GTGATGGCTCAAGTACATGAGAG	[2:3:9:12]	F:CACCAAGTGGAATGTGGTTAGA<br>R:CCATGTGAGATACTGTGCTTCC
	chr15:+38358573	GGCGGAGCCCATGTACATGAAGG	[4:5:6:12]	F:CACCTCTGCTTGTTCTCTAACC<br>R:CTTCCCACCCACACTTCTATC

Table S4 . Alleles from the F1 generation rabbits.

Intercoross-F0 (Male*Female)	F1 generation No.	Target site mutations	Sequenced cononies	Phenotype
F0-7* F0-4	F1-1	Del 52 bp	8	+
		Del 57 bp	12	
	F1-2	WT	15	-
		Del 54 bp	5	
	F1-3	WT	20	-
	F1-4	WT	11	+
		Del 52 bp	9	
	F1-5	WT	20	-
	F1-6	WT	14	-
		Del 57 bp	6	
	F1-7	WT	6	-
		Del 57 bp	14	
F0-5* F0-8	F1-8	WT	13	+
		Del 79 bp	7	
	F1-9	WT	12	+
		Del 79 bp	8	
	F1-10	WT	20	-
	F1-11	WT	7	+
		Del 79 bp	13	
	F1-12	WT	20	-
	F1-13	WT	9	+
		Del 79 bp	11	
	F1-14	WT	9	+
		Del 79 bp	13	
	F1-15	WT	20	-
	F1-16	WT	20	-
	F1-17	WT	12	+
		Del 79 bp	9	
F0-7* F0-9	F1-18	WT	12	+
		Del 52 bp	10	
	F1-19	WT	11	+
		Del 52 bp	9	
	F1-20	WT	13	+
		Del 52 bp	7	
	F1-21	WT	13	+
		Del 54 bp	7	
	F1-22	WT	8	+
		Del 56 bp; insert 3 bp	12	
	F1-23	WT	15	+
		Del 52 bp	5	
	F1-24	WT	13	+
		Del 56 bp; insert 3 bp	7	

Note: WT: wild type allele; Del: deletion; insert: insertion.

3. Original file for Figures

3.1 Authors' original file for figure 1C

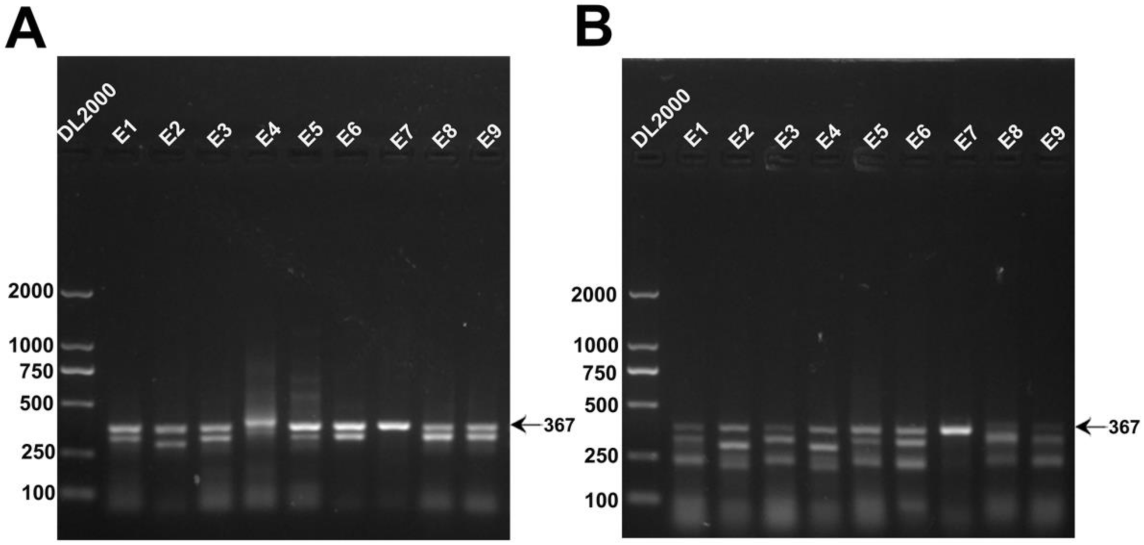

3.2 Authors' original file for figure 2B

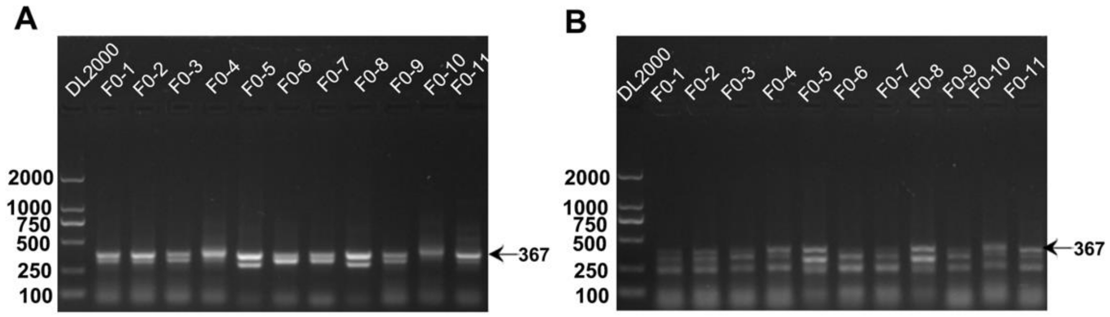

3.3 Authors' original file for figure 3C and 3D

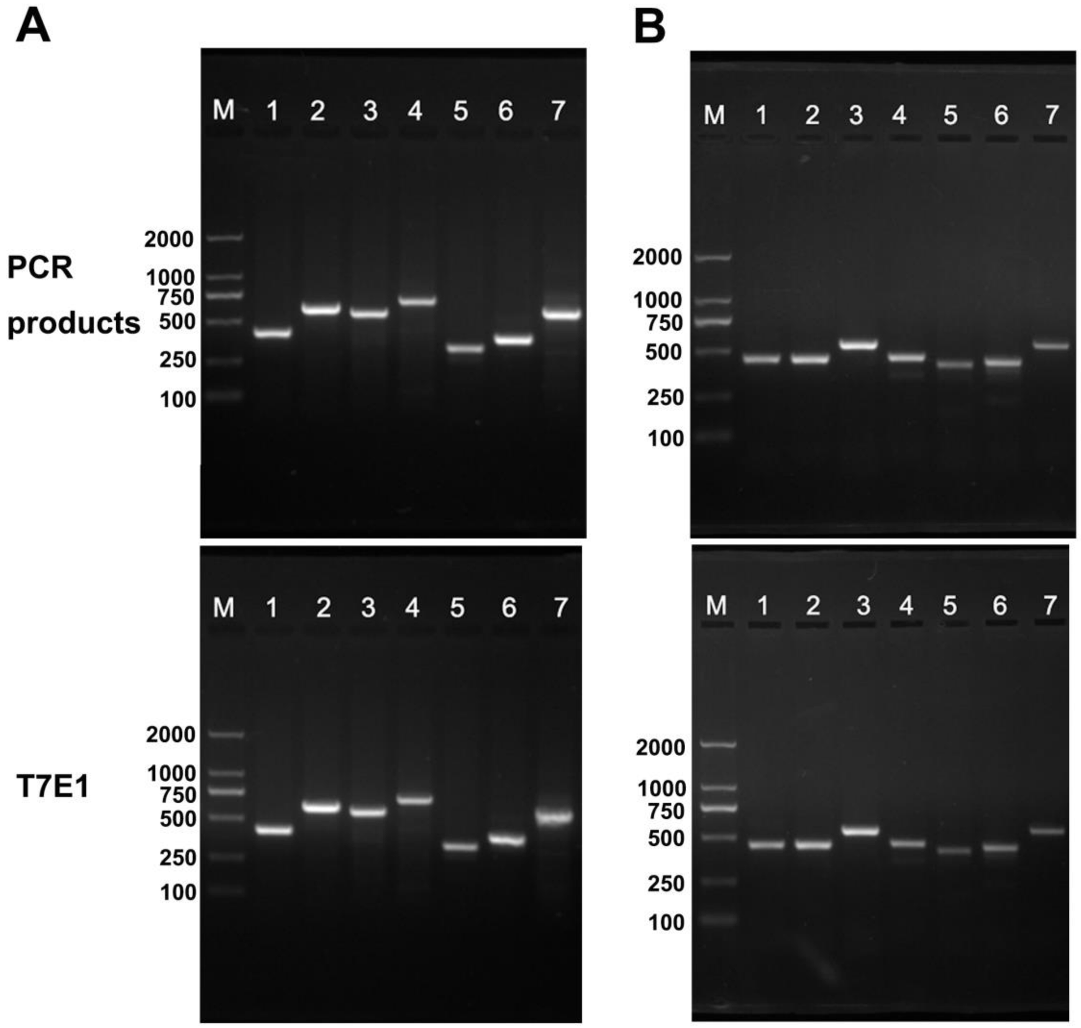

3.4 Authors' original file for figure 5A

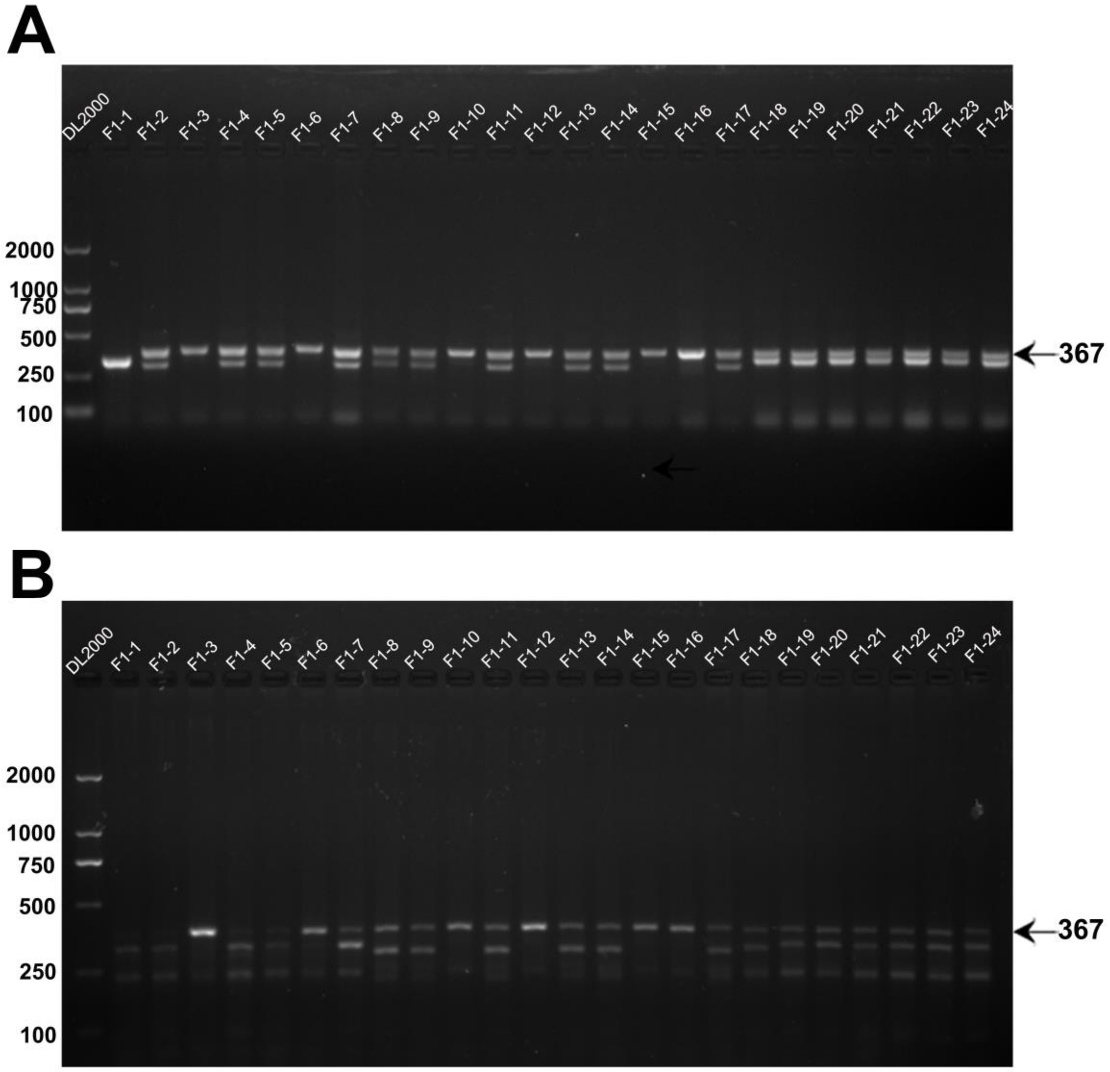

Supplement: Supplementary Information [file srep22024-s1.pdf]
